# GABA_A_-Receptor Agonists Limit Pneumonitis and Death in Murine Coronavirus-Infected Mice

**DOI:** 10.3390/v13060966

**Published:** 2021-05-23

**Authors:** Jide Tian, Blake Middleton, Daniel L. Kaufman

**Affiliations:** Department of Molecular and Medical Pharmacology, University of California, Los Angeles, CA 90095, USA; bmiddleton@mednet.ucla.edu

**Keywords:** GABA, GABA-receptors, mouse hepatitis virus (MHV), therapy, COVID-19

## Abstract

There is an urgent need for new approaches to limit the severity of coronavirus infections. Many cells of the immune system express receptors for the neurotransmitter γ-aminobutyric acid (GABA), and GABA-receptor (GABA-R) agonists have anti-inflammatory effects. Lung epithelial cells also express GABA-Rs, and GABA-R modulators have been shown to limit acute lung injuries. There is currently, however, no information on whether GABA-R agonists might impact the course of a viral infection. Here, we assessed whether clinically applicable GABA-R agonists could be repurposed for the treatment of a lethal coronavirus (murine hepatitis virus 1, MHV-1) infection in mice. We found that oral GABA administration before, or after the appearance of symptoms, very effectively limited MHV-1-induced pneumonitis, severe illness, and death. GABA treatment also reduced viral load in the lungs, suggesting that GABA-Rs may provide a new druggable target to limit coronavirus replication. Treatment with the GABA_A_-R-specific agonist homotaurine, but not the GABA_B_-R-specific agonist baclofen, significantly reduced the severity of pneumonitis and death rates in MHV-1-infected mice, indicating that the therapeutic effects were mediated primarily through GABA_A_-Rs. Since GABA and homotaurine are safe for human consumption, they are promising candidates to help treat coronavirus infections.

## 1. Introduction

While GABA-Rs are well known for their role in neurotransmission in the central nervous system, these receptors are also found on some cell types in the periphery, including cells of the immune system and lung epithelial cells. The biological roles of GABA-Rs on immune cells are not yet well understood, but there is a growing body of evidence that the activation of these receptors has immunoregulatory actions. There are two types of GABA-Rs that are encoded by unrelated gene families: GABA_A_-Rs are heteropentamers of 19 possible subunits that form fast-acting chloride channels [1] and GABA_B_-Rs are heterodimers that form a slow-acting G-protein coupled receptor [2]. Rodent and human macrophages and dendric cells express both GABA_A_-Rs and GABA_B_-Rs, and GABA and GABA_A_-R-specific agonists inhibit their inflammatory activity [3,4,5,6,7]. T cells have been shown to express GABA_A_-Rs, but there is no functional evidence of their expression of GABA_B_-Rs [6,8,9,10,11,12]. The oral administration of GABA or the GABA_A_-R agonist homotaurine inhibits autoreactive Th1 and Th17 cells while promoting CD4^+^ and CD8^+^ Treg responses [10,11,13], ameliorates autoimmune disease in mouse models of type 1 diabetes (T1D), multiple sclerosis, and rheumatoid arthritis, and also limits inflammation in murine type 2 diabetes [3,10,11,14,15]. The GABA_B_-R agonist baclofen also has anti-inflammatory actions and has been shown to inhibit arthritis, dermatitis, and T1D in rodent models [16,17,18]. Human immune cells also express GABA-Rs, and GABA inhibits secretion of IL-6, TNF, IL-17A, CXCL10/IP-10, CCL4, CCL20, and MCP-3 from anti-CD3- stimulated PBMC from T1D patients [8]. The ability of GABA-R agonists to inhibit the production of different inflammatory factors is of potential interest for helping to treat COVID-19 since high serum levels of some of these inflammatory factors are associated with the development of severe COVID-19 [19,20,21,22].

Lung epithelial cells also express GABA-Rs. GABA, a GABA_B_-agonist (baclofen), and GABA_A_-R-positive allosteric modulators (PAMs) have been shown to reduce inflammation and improve alveolar fluid clearance and lung functional recovery in different rodent models of acute lung injury (e.g., endotoxin or ventilator-induced injuries [23,24,25,26,27,28,29,30]), as well as limit pulmonary inflammatory responses and improve clinical outcomes in ventilated human patients [31,32,33]. Treatment with GABA_A_-R PAMs can reduce the number of macrophages and the levels of inflammatory cytokines in bronchoalveolar lavage fluid and limit inflammatory responses by rodent and human macrophages [29,34,35,36,37,38]. GABA application reduces the secretion of inflammatory factors from lipopolysaccharide-stimulated human bronchial epithelial cells in vitro [24]. Finally, GABA can inhibit platelet aggregation [39], which could have clinical potential because pulmonary thrombosis often occurs in critically ill COVID-19 patients [40].

The aforementioned actions of GABA-R modulators on immune cells, lung alveolar epithelial cells and platelets make them potential candidates for limiting the severe pneumonia and lung damage that can occur due to coronavirus infection. Currently, however, there is no information on whether treatment with a GABA-R agonist modulates the clinical outcome of any viral infection. 

Mouse hepatitis virus (MHV)-1 is a pneumotropic beta-coronavirus that is widely used as a safe model of SARS-CoV and SARS-CoV-2 infection [41,42,43,44]. Intranasal inoculation with ≥5 × 10^3^ plaque-forming units (PFU) of MHV-1 in A/J mice induces acute pneumonitis and acute respiratory distress syndrome (ARDS) with a high lethality rate. The MHV-1-infected mice develop clinical symptoms and pathological features similar to those in severely ill COVID-19 patients, including high levels of pulmonary edema, pneumonitis, dense inflammatory infiltrates, hyaline-like membranes, and fibrin deposits, accompanied by loss of body weight and respiratory distress [41,42,43,44]. Here, we assessed whether GABA-R agonists had therapeutic potential for treating this model of severe COVID-19. We found that GABA treatment reduced the pneumonitis, disease severity and death rate when given before or after the onset of symptoms, and that these effects were mediated through activating GABA_A_-Rs. We also observed that GABA treatment significantly reduced viral loads and pathological changes in their lungs. We discuss the possible mechanisms by which GABA_A_-R activation attenuates disease in MHV-1-infected mice.

## 2. Results

**Figure 1:**
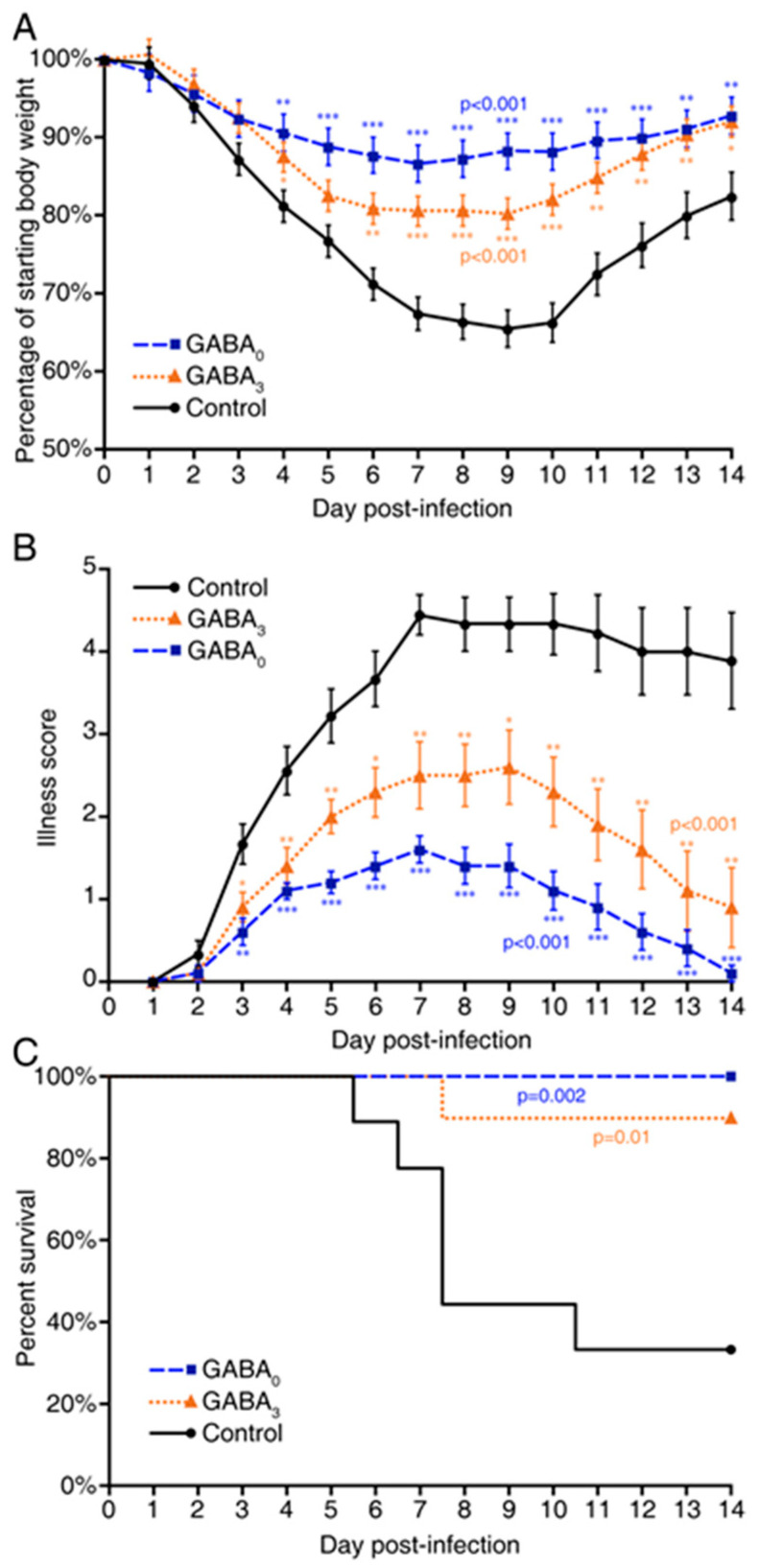
GABA treatment limits disease progression and death rate in MHV-1-infected mice. A/J mice were inoculated with MHV-1 intranasally and immediately placed on plain water (control, solid black line) or water containing 20 mg/mL GABA (GABA0, dashed blue line), or given plain water for 3 days post-infection and then placed on water containing 20 mg/mL GABA (GABA3, orange dotted line) for the remaining observation period: (**A**) Daily changes in mean % ± SEM body weight post-infection (% of day 0). GABA0 and GABA3 vs. control *p* < 0.001. GABA0 vs. GABA3 *p* = 0.159. Mean weight ratio profiles were computed under a repeated measure (mixed) analysis of variance model (RM ANOVA); (**B**) The animals were scored daily for the severity of their illness as detailed in Materials and Methods. The data shown are the mean illness scores +/− SEM of each group from two separate experiments. Overall *p* < 0.001 for GABA0 and GABA3 vs. control, and *p* = 0.042 for GABA0 vs. GABA3 using the Kruskal–Wallis test. Individual *p* values indicated as * *p* < 0.05, ** *p* < 0.01, *** *p* < 0.001; (**C**) Daily percent of surviving mice in each group. GABA0 and GABA3 vs. control *p* = 0.002 and *p* = 0.01, respectively by log-rank test. GABA0 vs. GABA3 *p* = 0.32. *n* = 9 mice in the control group, 10 mice in each GABA-treated group. Individual mouse identification number was used as the random effect. Data shown are from two separate studies with 4–5 mice/group.

In our first study, A/J mice were inoculated intranasally with MHV-1 (5 × 10^3^ PFU) and then randomized to receive plain water (controls) or GABA through their drinking water (20 mg/mL, an effective therapeutic dose in T1D mice [13]) for the entirety of the observation period. Another group of MHV-1-inoculated mice received plain water for three days, by which time they displayed clear signs of illness, and were then placed on GABA-containing water for the rest of the observation period. Following MHV-1 inoculation, the mice receiving plain water began to progressively lose body weight each day. By day 5, this control group had lost an average of 23% of their weight (Figure 1A and Supplemental Figure S1), as expected [41,42,43,44]. At this time point, the mice that had been given GABA had lost significantly less body weight; 11% and 17% weight loss for mice given GABA immediately and three-days post-infection, respectively (Figure 1A and Supplemental Figure S1).

In terms of illness, MHV-1-infected control mice began to display signs of illness two days post-infection and rapidly became severely ill thereafter, with their illness peaking around day 7 post-infection (Figure 1B and Supplemental Figure S2). In contrast, the mice receiving GABA immediately after MHV-1 inoculation developed only mild illness. Illness scores in the mice given GABA at 3 days post-infection was also significantly reduced compared to that in the control group (Figure 1B and Supplemental Figure S2). Thus, GABA treatment immediately after MHV-1 infection, or 3 days later when the clinical signs of the disease were apparent, reduced the severity of the disease. 

GABA treatment led to significantly reduced death rates in MHV-1-infected mice. Six days after inoculation, the mice in the control group began to succumb to their illness and only 3/9 mice survived on day 14 post-infection (Figure 1C). In contrast, all of the mice (*n* = 10) given GABA starting immediately after MHV-1 inoculation survived (Figure 1C). Of the mice that began GABA treatment at 3 days post-infection, 9/10 mice survived.

**Figure 2:**
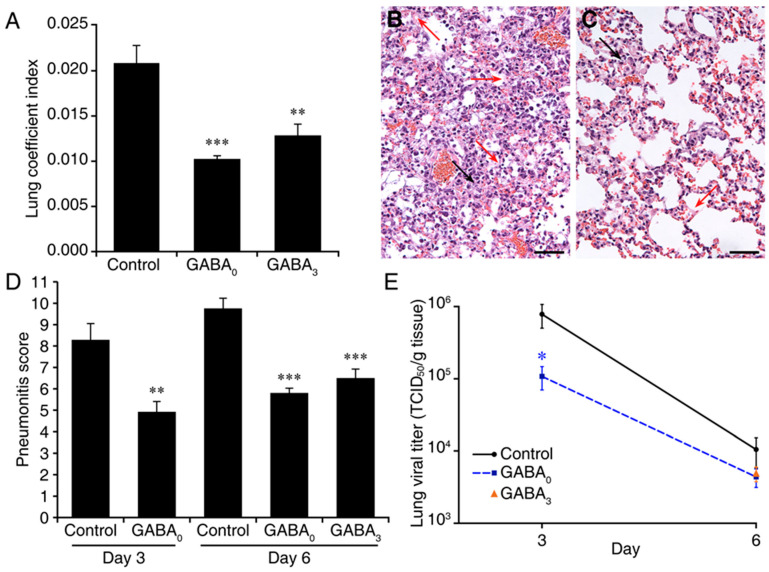
GABA treatment reduces pneumonitis and viral load in MHV-1-infected mice: (**A**) GABA treatment reduces lung coefficient index. Mice were inoculated with MHV-1 and placed on plain water (control) or water containing GABA (20 mg/mL) immediately (GABA0) or 3 days post-infection (GABA3). The lungs were harvested and weighed when an animal became moribund or at 14 days post-infection. Data shown are the mean lung coefficient index ± SEM for each group from two separate studies. *** *p* < 0.001 and ** *p* < 0.01 for GABA0 and GABA3 (respectively) vs. control water-treated group by Student’s *t*-test; (**B**,**C**) Histopathological features in the lungs of untreated and GABA-treated mice at 6 days post-MHV-1 infection. Representative images of H&E-stained lung sections (magnification ×200) from (**B**) untreated mice and (**C**) GABA-treated (beginning immediately following inoculation) mice. Red arrows point to hyaline-like membranes and black arrows indicate local consolidation. The scale bar is 50 µm; (**D**) Quantitative analysis of the degrees of pneumonitis in the lungs based on the number of hyaline-like membranes, % of pulmonary areas with obvious inflammatory infiltrates in lung parenchyma, and the % of area with inflammatory consolidation (as described in Materials and Methods). Data are expressed as the mean pneumonitis score ± SEM of each group (*n* = 5 mice per group per time point in two independent experiments); (**E**) Kinetics of MHV-1 replication in the lungs. Mice were inoculated with MHV-1 and immediately placed on plain water (control) or water containing GABA (20 mg/mL) and 3 or 6 days later, their lungs were harvested for determination of viral load. Concurrently, another group of MHV-1-inoculated mice was given water containing GABA (20 mg/mL) beginning 3 days post-infection, and the viral load in their lungs was determined 6 days post-infection. The data shown are the mean TCID50/g of lung tissue ± SEM at the indicated days. GABA0 mice (blue square symbol) received GABA immediately after inoculation and GABA3 mice (orange triangle symbol) received GABA beginning 3 days post-infection. *n* = 5 mice per group at each time point. * *p* < 0.05 by Student’s *t*-test.

The lung coefficient index (the ratio of lung weight to total body weight) reflects the extent of edema and inflammation in the lung. The lung coefficient index of mice that were given GABA immediately after MHV-1 infection was 49% of that of control mice (*p* < 0.001, Figure 2A and Supplemental Figure S3). The lung coefficient index in the mice receiving GABA treatment beginning 3 days post-infection was 62% of that in the control mice (*p* < 0.01, Figure 2A and Supplemental Figure S3). Consistent with the scores for illness, histological evaluation of lung sections from GABA-treated mice (immediately after MHV-1 inoculation) revealed reduced infiltrates, hyaline-like membrane formation and hemorrhage in the alveoli at 3 days post-infection, relative to that in control MHV-1-inoculated mice (Figure 2B,C). Quantitative analysis revealed that the pneumonitis scores in the mice that received GABA immediately after MHV-1 inoculation and examined 3 or 6 days later were significantly less than that in the respective control mice (Figure 2D and Supplemental Figure S4). Similarly, mice that were given GABA 3 days after inoculation also displayed reduced pneumonitis scores relative to control mice when examined 6 days post-infection (Figure 2D and Supplemental Figure S4). Thus, GABA treatment limited the MHV-1-induced lung damage in A/J mice.

We next assessed whether the reduced severity of MHV-1 induced disease in GABA-treated mice could be due in part to reduced virus production. A/J mice were inoculated with MHV-1 and given plain drinking water, or water containing GABA for the remainder of the study. Mice from these groups were euthanized 3 or 6 days post-infection and the viral load in their lungs was determined. Concurrently, another group of MHV-1 inoculated mice was given water containing GABA beginning at 3 days post-infection and the viral load in their lungs was determined 6 days post-infection. At 3 days post-infection, the mean viral load in the lungs of mice given plain water was about 7-fold higher than that in mice given GABA immediately after infection (*p* < 0.05, Figure 2E). Thereafter, the viral load in the lungs declined as expected, and by day 6 post-infection, the viral load in the lungs of control mice was about twice that in the lungs of mice given GABA immediately or 3 days post-infection, although these differences were not statistically significant (Figure 2E). Thus, early GABA treatment reduced viral loads in the lungs of mice.

**Figure 3:**
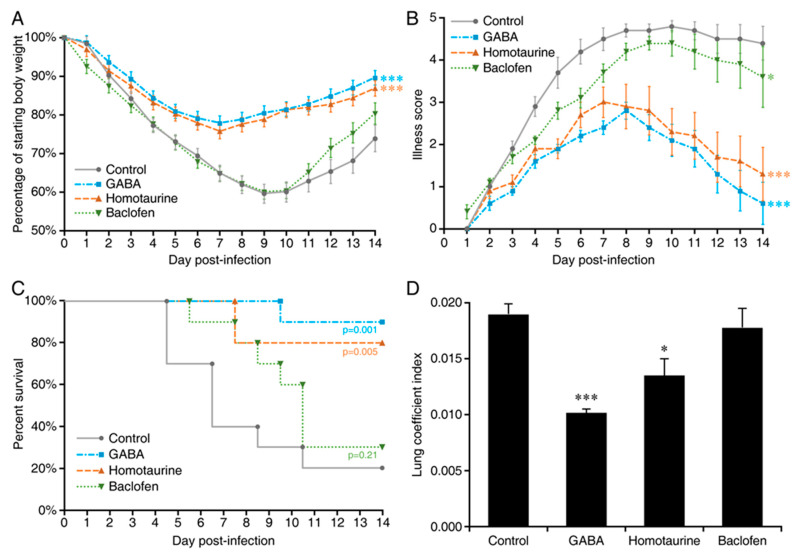
Effect of GABAA-R vs. GABAB-R agonists in MHV-1-infected mice. Mice were inoculated with MHV-1 (5 × 10^3^ PFU) and immediately given plain drinking water, or water containing GABA (2 mg/mL), homotaurine or baclofen. The average daily water consumption per mouse in each group is shown in Supplemental Figure S8: (**A**) Daily changes in mean % ± SEM of body weights post-infection (% of day 0), *** *p* < 0.001 vs. control, computed by a RM ANOVA model; (**B**) Daily scores for the severity of their illness. The data shown are the mean illness scores ± SEM for each group. *p* values are indicated for each treatment vs. the control as calculated by the Kruskal–Wallis test. * *p* < 0.05, *** *p* < 0.001; (**C**) Daily percent of surviving mice in each group. Indicated *p* values vs. the control were calculated by the log-rank test; (**D**) Lung coefficient indexes. The lungs were harvested and weighed when an animal became moribund or at 14 days post-infection. The data shown are the mean lung coefficient index ± SEM for each group. * *p* < 0.05, *** *p* < 0.001 vs. the control water treated group by Student’s *t*-test. For all studies, *n* = 10 mice/group from two separate experiments.

Finally, we asked whether GABA’s therapeutic effects were mediated through GABA_A_-Rs, GABA_B_-Rs, or both GABA-R subtypes. A/J mice were inoculated with MHV-1 and given plain water or water containing GABA (2 mg/mL), a clinically applicable GABA_A_-R-specific agonist (homotaurine, 0.25 mg/mL), or a GABA_B_-R-specific agonist (baclofen, 0.25 mg/mL). We found that treatment with GABA or homotaurine significantly reduced the body weight loss (Figure 3A and Supplemental Figure S5), disease scores (Figure 3B and Supplemental Figure S6), death rate (Figure 3C), and lung coefficient index in mice (Figure 3D and Supplemental Figure S7). Baclofen displayed a slight but significant ability to reduce illness scores; however, it did not significantly decrease the body weight loss, death rate, and lung coefficient index in these mice relative to that of untreated controls. Thus, GABA’s therapeutic effects are primarily mediated through GABA_A_-Rs. 

## 3. Discussion

While GABA-R activation has shown promising beneficial effects in animal models of autoimmune disease and acute lung injury, as well as in ventilated patients, there is no information as to whether these properties would impact an acute viral infection. Indeed, we had anticipated that early treatment with GABA following MHV-1 infection could be deleterious by reducing or delaying innate immune responses that help control the infection. On the contrary, GABA treatment immediately after MHV-1 infection very effectively prevented the development of severe illness and death. Moreover, GABA treatment beginning after the appearance of symptoms rapidly curtailed disease progression. GABA-treated mice also had smaller lung coefficient indexes (indicative of less edema and inflammation), and their lungs had significantly less inflammatory consolidation, as well as fewer hyaline-like membrane formations. Thus, GABA-R activation can limit a very acute and highly lethal viral respiratory infection—a property that heretofore was unknown.

There are a number of different biological processes through which GABA treatment may have ameliorated the severity of MHV-1-induced disease: (1) GABA and homotaurine inhibit macrophage and dendritic cell inflammatory activities [3,4,5,16,17,36]. Likewise, GABAA-R PAMs reduce the number of macrophages in bronchoalveolar lavage fluid, lung secretion of inflammatory cytokines, and inflammatory responses by rodent and human macrophages [29,34,35,36,37,38]. GABAA-R agonists also inhibit activated Th17 and Th1 responses and promote CD4^+^ and CD8^+^ Tregs [10,11,13]; however, since adaptive immune responses take about a week to arise, these abilities are unlikely to have contributed to GABA’s ability to attenuate disease very soon after MHV-1 infection. These effects on adaptive immune responses may be relevant for treating COVID-19 which has a longer disease course and in which high levels of circulating Th1, Th17, and Th2-secreted proteins are associated with severe illness [19,20]; (2) GABA treatment may have limited deleterious inflammatory responses to the infection by reducing viral loads in the lungs (see below); (3) GABA and GABA_A_-R PAMs reduce inflammation and improve alveolar fluid clearance and lung functional recovery in animal models of acute lung injury [23,24,25,27,28,29,30] and in ventilated patients [31,32,33], and could have exerted similar actions in the MHV-1-infected mice; (4) GABA and GABA_A_-R agonist treatments increase macrophage autophagy [45]. In murine models of pneumatic bacterial infections, GABA_A_-R agonists reduced bacterial load and TNFα and IL-6 levels in the lungs, and protected the mice against illness [45]; (5) GABA inhibits platelet aggregation [39]. Thus, treatment with GABA_A_-R agonists may have led to better outcomes in MHV-1-infected mice through multiple and diverse pathways. 

Surprisingly, early GABA treatment reduced viral loads in the lungs of mice when measured near the time of peak viral load in this model [41,42,43], which may have contributed to the better outcomes observed in GABA-treated animals. Thus, GABA-Rs may provide a new druggable target capable of reducing coronavirus production. We can envision a number of ways that GABA may have limited viral load: (1) The lung alveolar cells of rodents and humans express GABA_A_-Rs [37,46]. While the activation of GABA_A_-R’s Cl^−^ channels on neurons leads to Cl^−^ influx and hyperpolarization, the activation of GABA_A_-Rs on ATII cells induces Cl^−^ efflux and greater membrane depolarization [37,46]. As coronaviruses promote Ca^2+^ influx to enhance their replication [47,48], the activation of ATII GABA_A_-Rs and the ensuing Cl^−^ efflux and membrane depolarization may limit the influx of extracellular Ca^2+^, making the cellular environment less conducive to viral replication; (2) Activation of GABA_A_-Rs on lung alveolar and large airway epithelial cells may have (i) altered the secretion of immune signaling molecules from infected cells, (ii) altered alveolar surfactant production/absorption, and/or (iii) altered inflammatory responses and autophagy [45] and, (iv) reduced the expression of the MHV-1 receptor CAECAM1 in ways that limited virus spreading and production. Further detailed studies are needed to evaluate whether these factors and/or others contribute to the observed reduction in viral load.

Our studies also revealed that the GABA_A_-R-specific agonist homotaurine treatment had essentially the same efficacy as GABA to reduce body weight loss, severe illness, lung coefficient index, and death in MHV-1-infected mice. In contrast, the GABA_B_-R specific agonist baclofen displayed a slight but significant ability to reduce illness scores; however, the weight loss, lung coefficient indexes, and death rate in these mice were similar to that of untreated controls. Evidently, GABA’s therapeutic effects are primarily mediated through GABA_A_-Rs which are present on many types of immune cells [49] and lung epithelial cells [37,46]. Further granular studies are needed to understand the possible roles that the GABA_A_-Rs on these cell types play in our observations.

Oral GABA treatment was tested in hundreds of epilepsy patients for its ability to reduce seizures [50,51,52]. It had no clinical benefit, probably because it is unable to cross the blood–brain barrier, but there were no adverse effects in these long-term studies. A recent phase Ib GABA oral dosing study indicated that GABA is safe for consumption at up to 6 g/day [53]. The human equivalent dose (calculated as per [54]) of the GABA used in the Figure 3 experiments (2 mg/mL with ≈2.5 mL/day consumed per mouse, Supplemental Figure S8) is 688 mg/day for a 70 kg person, a dose that is available in nutraceutical formulations. Additionally, homotaurine was tested in a 78-week-long large phase III clinical trial for Alzheimer’s disease and while it was not effective, it had an excellent safety record [55,56,57]. The human equivalent dose of that used in our studies is less than that which was administered in this clinical trial [55]. Both GABA and homotaurine are inexpensive, stable at room temperature, and available worldwide, making them excellent candidates for clinical testing as adjunctive treatments for COVID-19.

Much remains to be learned about the mechanisms by which GABA_A_-R agonists protected MHV-1-infected mice from severe pneumonitis and whether these observations extend to SARS-CoV-2 infection in humans. Given that GABA and homotaurine can affect many biological processes and that viral infection is a very dynamic process, it is clear that GABA_A_-R agonist dosing needs to be carefully studied and optimized for different stages of coronavirus infection. Our observations provide a springboard for further investigations into whether targeting GABA_A_-Rs can provide new avenues to limit severe illness due to infection with SARS-CoV-2 and other novel coronaviruses.

## 4. Materials and Methods 

Mice. Previous studies of MHV-1-infected mice found that female A/J mice were especially sensitive to MHV-1 infection [41,42,43,44], and were therefore studied here as well. A/J mice (8 weeks in age) were purchased from the Jackson Laboratory, were housed in microisolator cages (4–5 mice/cage), and fed with a standard diet and water *ad libitum*. One week after arrival, they were inoculated with MHV-1. The mice were immediately randomized and treated (or not treated) with GABA, homotaurine or baclofen through their drinking water as described below. This study was carried out in accordance with the recommendations of the Guide for the Care and Use of Laboratory Animals of the National Institutes of Health. The protocols for all experiments using vertebrate animals were approved by the Animal Research Committee at UCLA (Protocol ID: ARC #2020-122; Date 25 August 2020–24 August 2023) and were carried out in compliance with the ARRIVE guidelines.

Reagents. GABA (stock #A2129), homotaurine (stock #A76109), and baclofen (stock #B5399) were purchased from Millipore-Sigma (St. Louis, MO, USA). 

Virus. MHV-1, DBT cells, and HeLa-CECAM1 were generously provided by Dr. Stanley Perlman (University of Iowa). MHV-I virus was prepared and titered as previously described [41,42,43,44].

Viral infection and GABA-R agonist treatment. At 9 weeks of age, A/J mice were anesthetized and inoculated intranasally with 5 × 10^3^ PFU MHV-1 in 50 µL cold Dulbecco’s modified Eagle’s medium (DMEM). The mice were immediately randomized and provided with plain water (controls) or water bottles that contained GABA (2 or 20 mg/mL as indicated, as in [3,13]), homotaurine (0.25 mg/mL, as per [10]) or baclofen (0.25 mg/mL as per [18]) for the entirety of the observation period. Some MHV-1-inoculated mice received plain water for 3 days, by which time they displayed signs of illness, and then were placed on GABA-containing water (20 mg/mL) for the rest of the observation period. Their body weights were measured daily up to 14 days post-infection. In addition, some MHV-1-infected mice were randomized and provided with plain water (control) or water containing GABA (20 mg/mL) immediately or 3 days post-infection. The mice were euthanized at day 3 or 6 days post-infection. Their right lungs were dissected for measurement of viral loads and their left lungs for histological examination.

Illness scoring. Individual mice were monitored daily for their illness development and progression which were scored on the following scale: (0) no symptoms; (1) slightly ruffled fur and altered hind limb posture; (2) ruffled fur and mildly labored breathing; (3) ruffled fur, inactive, moderately labored breathing; (4) ruffled fur, inactive, obviously labored breathing, hunched posture; (5) moribund or dead. 

The percent survival of each group of mice was determined longitudinally. Mice with a disease score of 5 were weighed, euthanized, and their lungs removed and weighed for calculation of lung coefficient index (the ratio of lung weight to total body weight, which reflects the extent of edema and inflammation in the lungs). On day 14 post-infection, the surviving animals were weighed, euthanized, and their lungs were removed and weighed for determination of the lung coefficient index.

Hematoxylin and eosin staining of lung sections. Their left lungs were fixed in 10% neutral buffered formalin and embedded in paraffin. Lung tissue sections (4 µm) were routine-stained with hematoxylin and eosin. Five images from each mouse were captured under a light microscope at 200 × magnification. The degrees of pathological changes were scored, based on the number of hyaline-like membranes, % of pulmonary areas with obvious inflammatory infiltrates in lung parenchyma, and the % of area with inflammatory consolidation within the total area of the section. The total numbers of hyaline-like membranes with, or without, cell debris or hyaline-like deposition in alveoli of the lung tissue section were scored as 0: none detectable; 1: 1–5; 2: 6–10; 3: >10. The areas of lung inflammation and hemorrhage in one lung section were estimated and the severity of inflammation and hemorrhage in the section was scored as 1: mild; 2: moderate; 3: marked; 4: severe. Accordingly, an inflammatory score in each mouse was obtained by % of lung areas × severity score. The areas of lung inflammatory consolidation were estimated in the lung section and scored as 1: ≤10%; 2: 11–25%; 3: 26–50%; 4; >50%. Finally, the pneumonitis score of individual mice = inflammation score + lung consolidation score + hyaline membrane score with a maximum score of 11.

Viral titers. Frozen lung samples were dounced into 1 mL of ice-cold DMEM with 10% fetal calf serum and homogenized with 1 mm glass beads using a Qiagen TissueLyser-LT at 50 Hz for 6 × 1 min. The viral titers in the supernatants were determined by endpoint dilution [58] in HeLa-CEACAM1 cells (85% confluent, 5 × 10^4^ cells/well) using the Spearman–Kärber formula [59] to calculate 50% tissue culture infectious dose (TCID_50_). 

Statistics. Statistical methods are described in each figure legend. A *p* value of <0.05 was considered to be statistically significant.

## Data Availability

The data presented in this study are available on request from the corresponding authors.

## Supplementary Materials

The following are available online at https://www.mdpi.com/article/10.3390/v13060966/s1, Figure S1: Longitudinal changes in body weights of individual mice. Figure S2: Longitudinal measurements of illness scores in individual mice. Figure S3: The lung coefficient index of individual mice. Figure S4: The pneumonitis index of individual mice. Figure S5: The dynamic changes in body weights of individual mice. Figure S6: Longitudinal measurements of illness scores in individual mice. Figure S7: The lung coefficient index of individual mice. Figure S8: Average daily consumption of drinking water.
