# Supplementary material for: GABAA-Receptor Agonists Limit Pneumonitis and Death in Murine Coronavirus-Infected Mice
Source: Viruses. 2021 May 23;13(6):966. doi: 10.3390/v13060966 (PMC8224554; doi:10.3390/v13060966)
Supplement: Supplementary file 1 [file viruses-13-00966-s001.zip › viruses-1213808-supplementary.pdf]

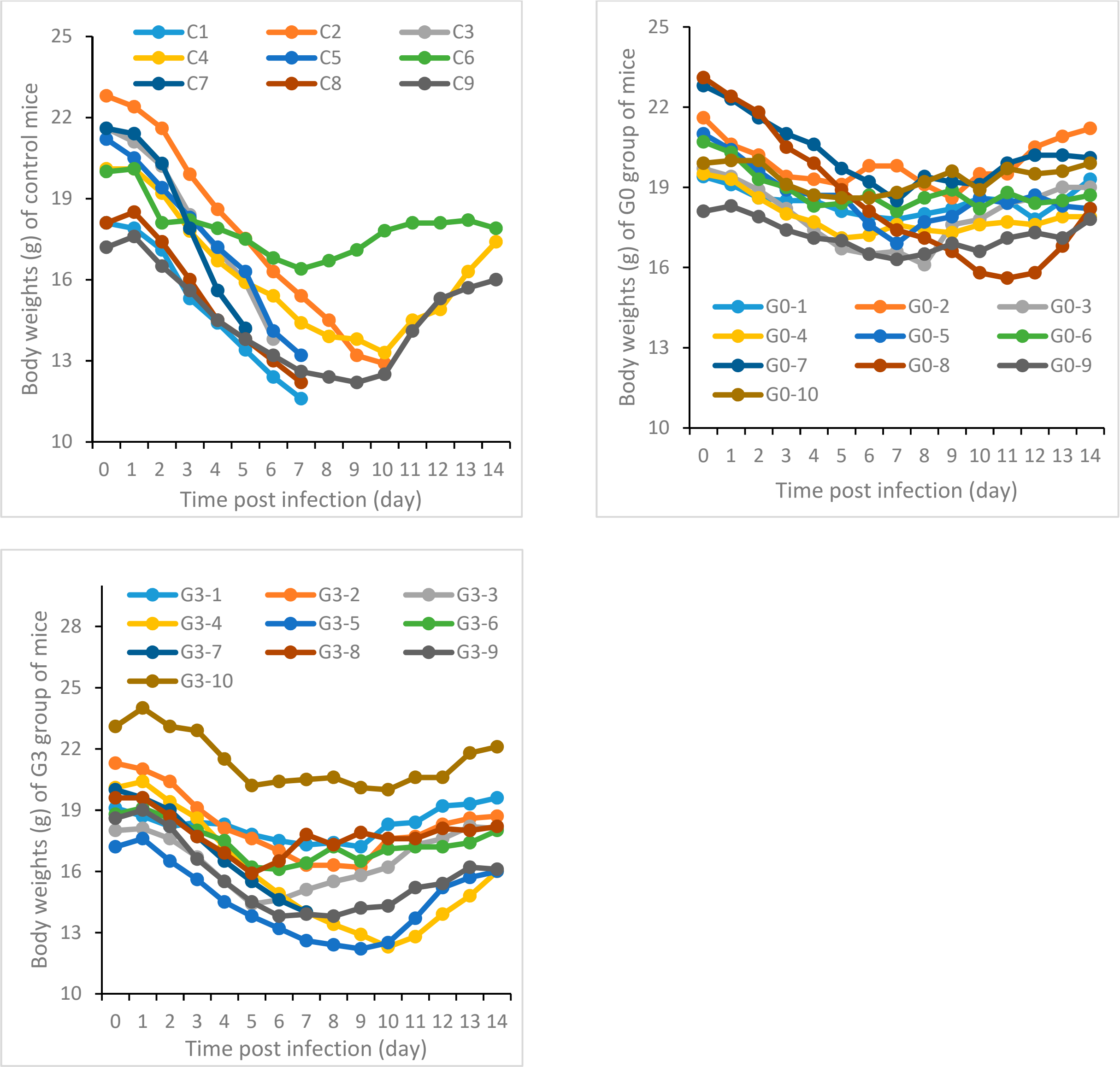

Supplementary Figure S1. Longitudinal changes in body weights of individual mice. All mice were infected with MHV-1 (5x10^3 PFU) at day 0 and their body weights were measured daily until their death or the end of the observation period (14 days post-infection). C: The control group of mice received plain water throughout the experimental period. G0: This group of mice received GABA (20 mg/ml) through their drinking water beginning on day 0. G3: This group of mice received plain water for three days post-infection and were thereafter given GABA treatment for the remainder of the study.

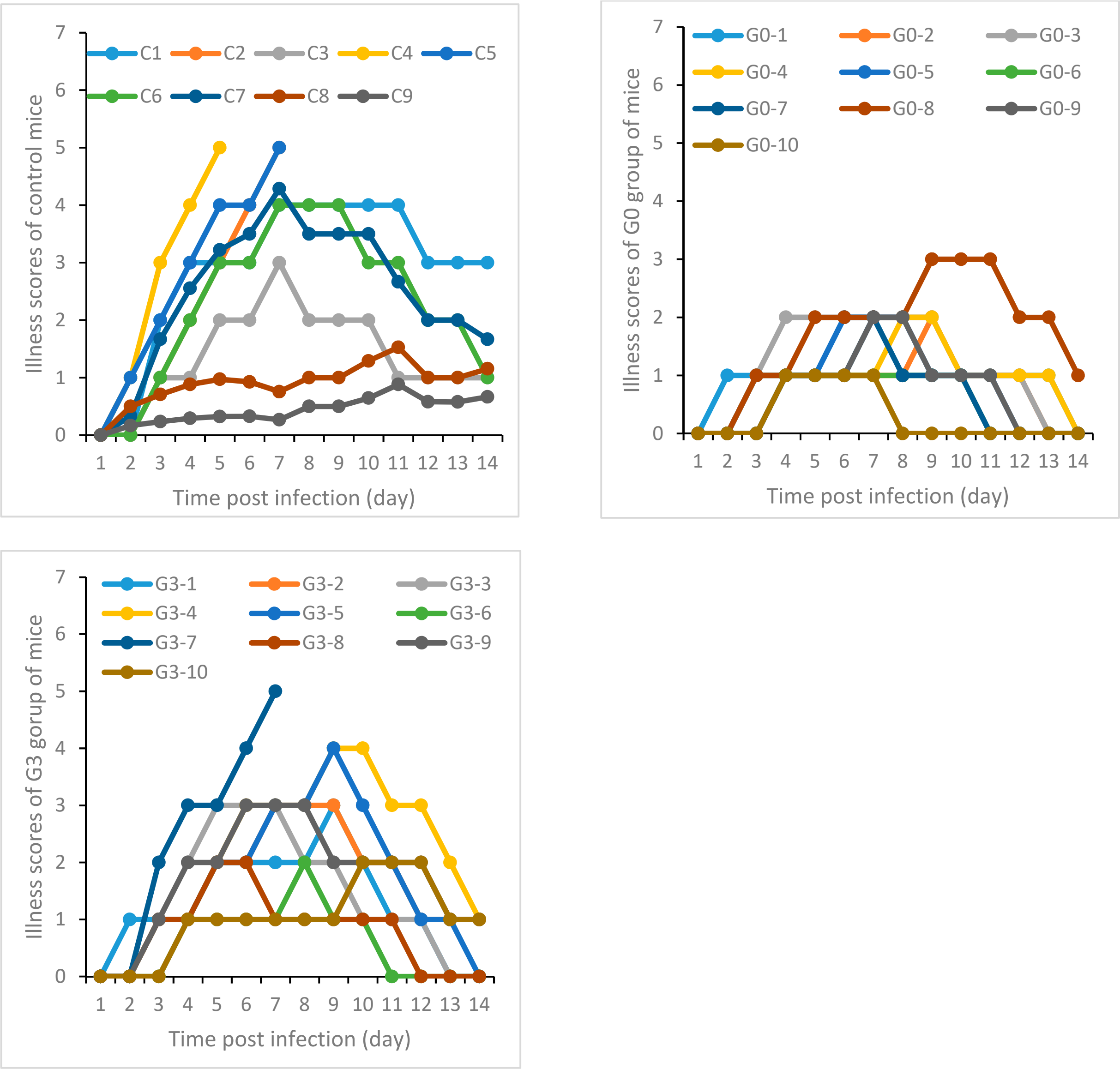

Supplementary Figure S2. Longitudinal measurements of illness scores in individual mice.

Following MHV-1 virus infection, each mouse was evaluated daily for the severity of its illness and scored as described Materials and Methods. C: The control group of mice received plain water throughout the experimental period. G0: This group of mice received GABA through their drinking water beginning on day 0. G3: This group of mice received plain water for three days and were then given GABA treatment for the remainder of the study.

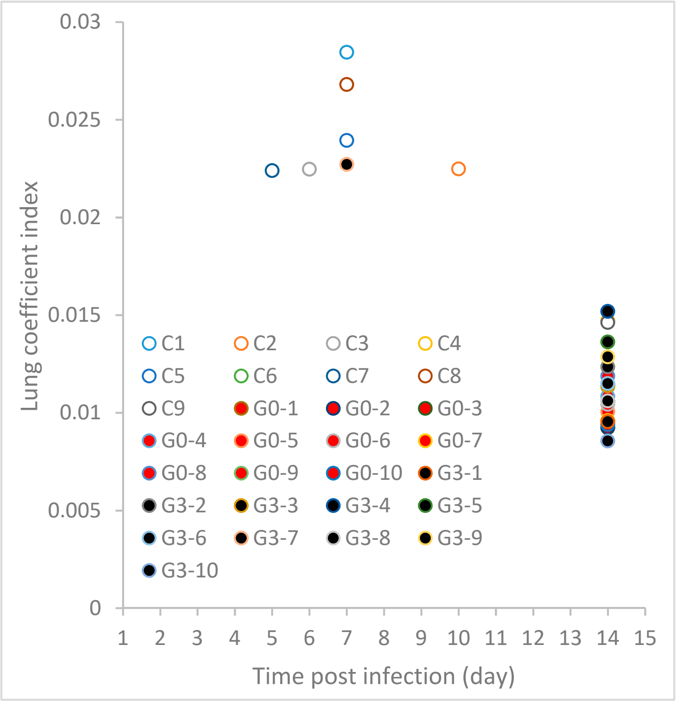

Supplementary Figure S3. The lung coefficient index of individual mice. Following MHV-1 infection, individual mice were monitored for clinical symptoms and their body weights were measured daily. Mice that reached an illness score of 5 or survived to the end of the observation period (14 days post-infection) were euthanized. Their lungs were dissected and weighed to calculate the lung coefficient index (the ratio of lung weight to body weight). C: The control group of mice received plain water throughout the experimental period (unfilled cycles). G0: This group of mice received GABA (20 mg/mL) through their drinking water (red-filled circles). G3: This group of mice received plain water for three days and were then given GABA treatment for the remainder of the study (black-filled circles).

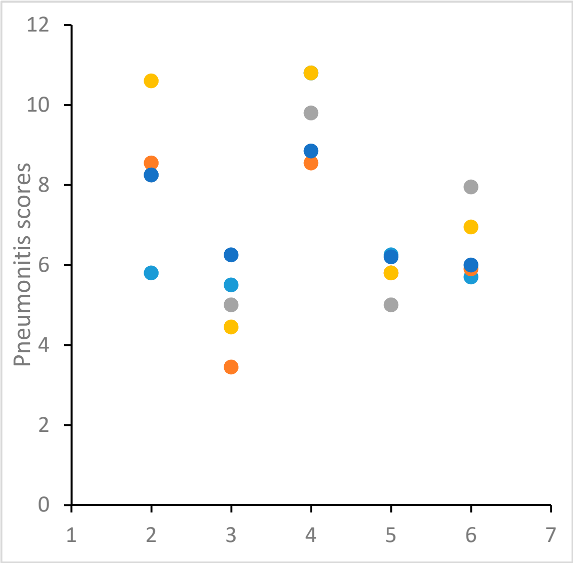

Supplementary Figure S4. The pneumonitis index of individual mice. Following MHV-1 infection mice were placed on plain water (control) or immediately placed on GABA water (GABA0). Another group of infected mice were placed on plain water and then switched to GABA-water at three days post-infection (GABA3). Three days post-infection, the lungs of five control mice (column 2) and five GABA0 mice (column 3) were harvested for analysis. Six days post-infection, the lungs of five control mice (column 4), G0 mice (column 5), and G3 mice (column 6) were harvested for histological examination and the severity of pneumonitis in individual lung tissue sections were scored, as described in Materials and Methods.

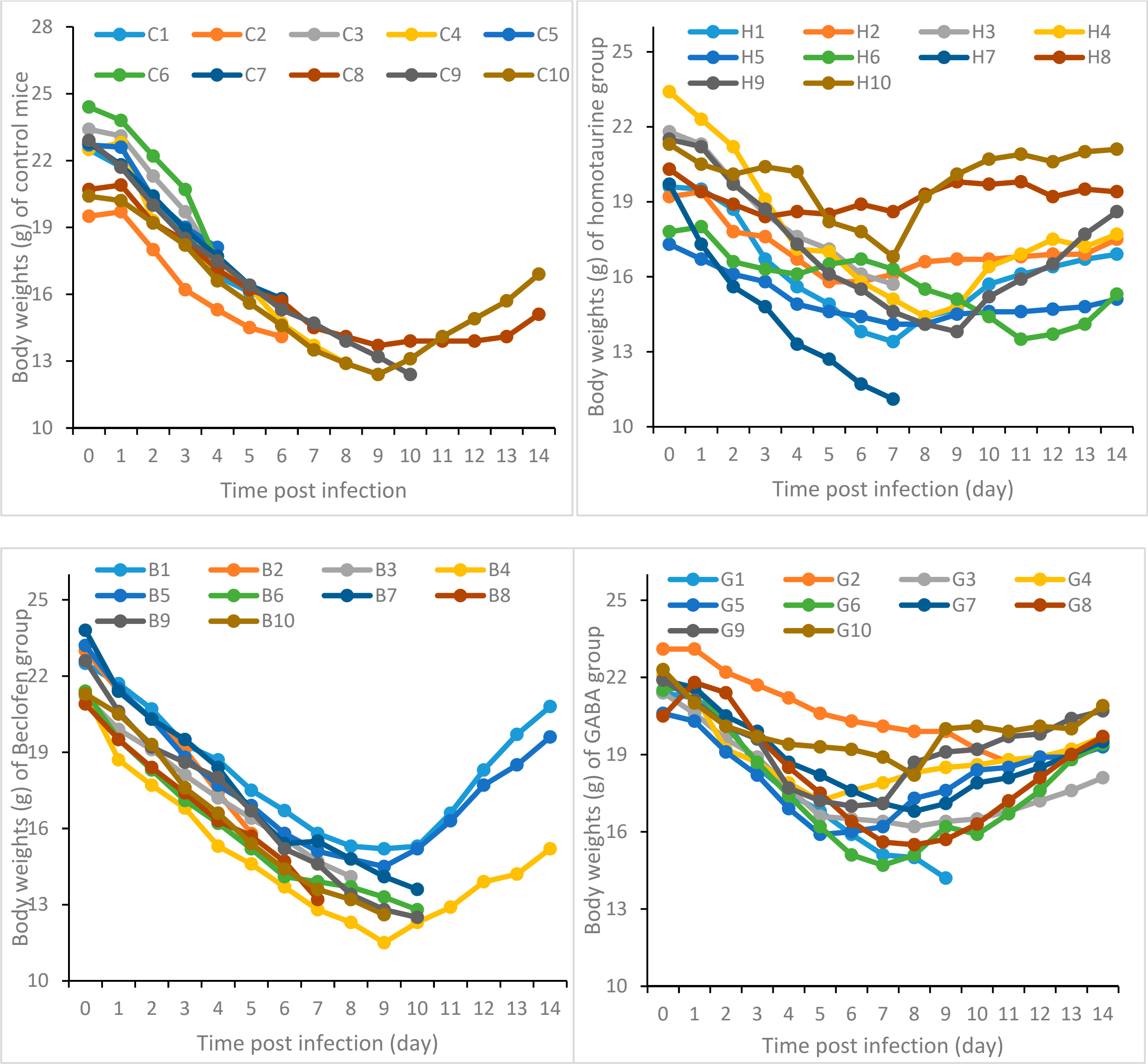

Supplementary Figure S5. The dynamic changes in body weights of individual mice. Following MHV-1 infection, the mice were immediately randomized to receive plain water (control) or drinking water containing GABA (2 mg/mL), homotaurine (0.25 mg/mL), or baclofen (0.25 mg/ml) throughout the observation period. Their body weights were measured daily. Mice were euthanized when they had an illness score of 5 or at the end of the observation period (14 days post-infection).

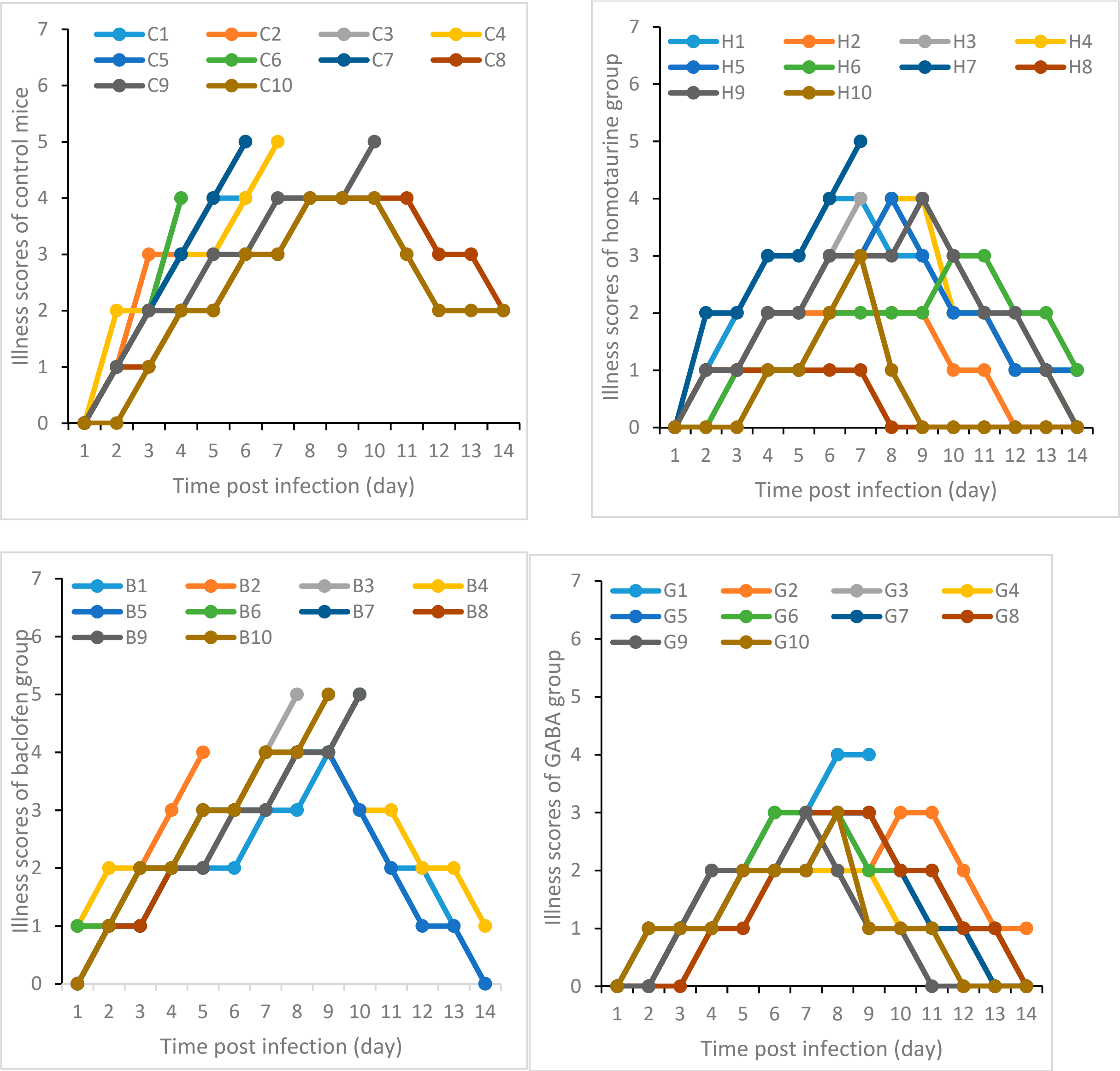

Supplementary Figure S6. Longitudinal measurements of illness scores in individual mice. Following MHV-1 infection, the mice were randomized to receive plain water, homotaurine, baclofen, or GABA through their drinking water (n=10 mice per group). Their clinical symptoms were monitored and illness severities were scored, as described in the section of Materials and Methods.

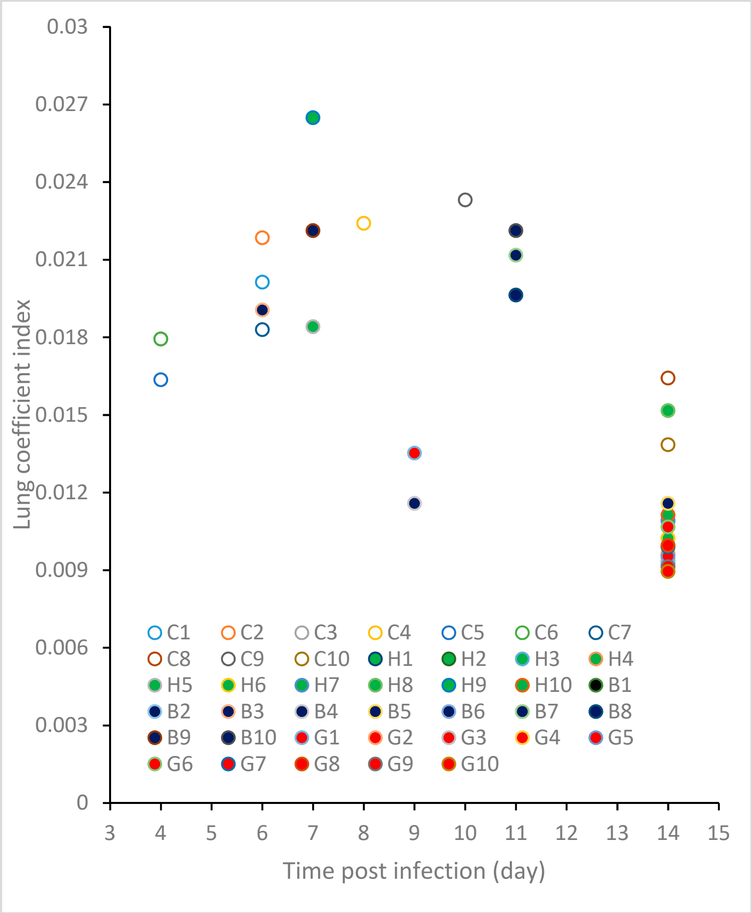

Supplementary Figure S7. The lung coefficient index of individual mice. Following MHV-1 infection, the mice were randomized to receive plain water (control, C unfilled circle), 0.25 mg/mL homotaurine (H, green-filled circle), 0.25 mg/mL baclofen (B, black-filled circle), or 2 mg/mL GABA (G, red-filled circle) treatment immediately. The mice were monitored for clinical symptoms and their body weights were measured daily. Mice that had an illness score of 5 or survived to the end of the observation period (14 days post-infection) were euthanized and their lungs were dissected and weighed to calculate the lung coefficient index.

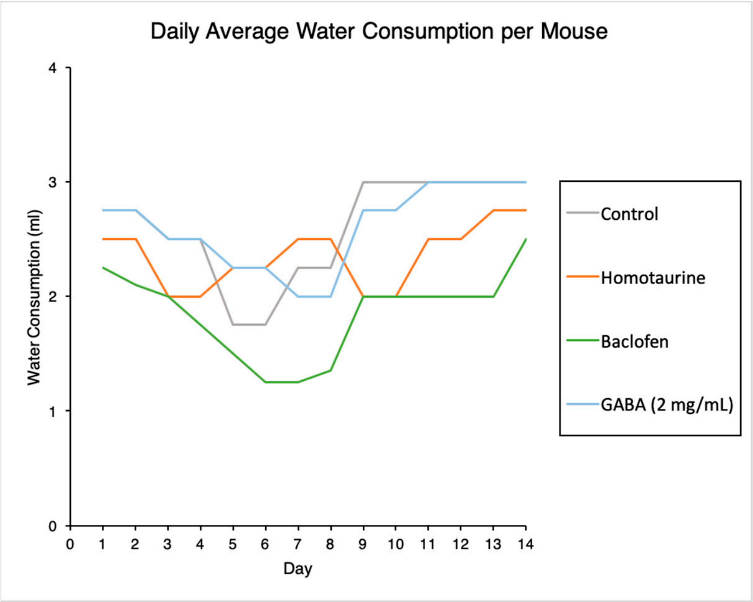

Supplementary Figure S8. Average daily consumption of drinking water. Mice were given plain water (control) or water containing homotaurine, baclofen, or GABA and allowed to drink the water *ad libitum*. We monitored how much water was consumed daily per cage and divided that by the number of surviving mice in the cage to calculate the average daily water consumption per mouse. For each treatment, we began with 5 mice/group and each group was housed in a single cage. We performed two independent studies and the data shown is the average daily water consumption (mL) per mouse post-infection.
